# Assessing the validity of a self-reported clinical diagnosis of schizophrenia

**DOI:** 10.1038/s41537-024-00526-5

**Published:** 2024-10-30

**Authors:** Grace E. Woolway, Sophie E. Legge, Amy J. Lynham, Sophie E. Smart, Leon Hubbard, Ellie R. Daniel, Antonio F. Pardiñas, Valentina Escott-Price, Michael C. O’Donovan, Michael J. Owen, Ian R. Jones, James T. R. Walters

**Affiliations:** Centre for Neuropsychiatric Genetics and Genomics, Division of Psychological Medicine and Clinical Neurosciences, School of Medicine, Cardiff University, Cardiff, UK

**Keywords:** Schizophrenia, Biomarkers

## Abstract

The increasing availability of biobanks is changing the way individuals are identified for genomic research. This study assesses the validity of a self-reported clinical diagnosis of schizophrenia. The study included 1744 clinically-ascertained participants with schizophrenia or schizoaffective disorder depressed-type (SA-D) diagnosed by self-report and/or research interview and 1453 UK Biobank participants with self-reported and/or medical record diagnosis of schizophrenia or SA-D. Unaffected controls included a total of 501,837 participants. We assessed the positive predictive values (PPV) of self-reported clinical diagnoses against research interview and medical record diagnoses. Polygenic risk scores (PRS) and phenotypes relating to demographics, education and employment were compared across diagnostic groups. The variance explained (r^2^) in schizophrenia PRS for each diagnostic group was compared to samples in the Psychiatric Genomics Consortium (PGC). In the clinically-ascertained participants, the PPV of self-reported schizophrenia for a research diagnosis of schizophrenia was 0.70, which increased to 0.81 after expanding the research diagnosis to schizophrenia or SA-D. In UK Biobank, the PPV of self-reported schizophrenia for a medical record diagnosis was 0.74. Compared to participants who self-reported, participants with a clinically-ascertained research diagnosis were younger and more likely to have a high school qualification. Participants with a medical record diagnosis in UK Biobank were less likely to be employed or have a high school qualification than those who self-reported. Schizophrenia PRS did not differ between participants that had a diagnosis from self-report, research diagnosis or medical records. Polygenic liability r^2^, for all diagnosis definitions, fell within the distribution of PGC schizophrenia cohorts. Self-reported measures of schizophrenia are justified in genomic research to maximise sample size and reduce the burden of in-depth interviews on participants, although within sample validation of diagnoses is recommended.

## Introduction

Schizophrenia is a severe mental health condition characterised by positive, negative, and disorganised symptoms as well as cognitive deficits^1^ and has a lifetime prevalence of 0.32% worldwide^2^. In research studies, schizophrenia diagnoses are now determined from various sources; for example, research interview and/or clinical note review, electronic heath records, or diagnoses can be based on a self-report of a clinical diagnosis made by a health professional. Methods combining diagnostic interviews and note reviews are considered the gold-standard for defining cases in research^3^, but are resource intensive and often associated with ascertainment biases; excluding those severely affected due to the lengthy interview but also milder cases that are not in contact in secondary health services leading to unrepresentative samples^4,5^.

Traditionally, genomics research has been founded on clinically-ascertained samples that provide a clinical or research interview diagnosis. The availability of large-scale biobanks only sets to increase in the coming years^6^ and holds great potential for psychiatric genomics, but the appropriateness and validity of these different diagnostic sources is unclear.

Diagnoses generated from medical records have been shown to have good concordance with research interview diagnoses^7,8^, with particularly high convergence seen in schizophrenia^9–11^. Ascertainment through medical records overcomes some of the practical limitations for participation, but still hinders representation by relying on records typically from secondary care^3^ and thus under-represents patients less severely affected who are less likely to be admitted to hospital. Alternatively, a self-reported diagnosis from a health professional could be one approach to improving representativeness and increasing sample size in genomic research, circumventing the need for a labour-intensive research interview. However, the validity of self-reported diagnoses is likely to differ between psychiatric disorders, contexts, and cultures. Research using self-reported medical diagnoses from large-scale genomic datasets such as 23andMe^12–17^, UK Biobank^13,16,18–21^ and the Million Veterans Programme^16,22^ are now common, but the reliability and validity of self-reported diagnoses is unclear^23^.

It is also unknown what impact different diagnostic methodologies such as self-report and medical records have on the outcome of genetic studies^24^. In order to enhance power in GWAS individuals with a self-reported diagnosis have been included^13,17^, and are likely to be increasingly so, despite some studies suggesting that individuals defined using minimal phenotyping approaches show genetic differences to participants who are strictly defined^23,25,26^. In one study, the effect sizes for schizophrenia polygenic risk scores (PRS) were reported to be smaller in samples where diagnoses are derived from electronic health records compared to clinically-ascertained case-control research cohorts in the Psychiatric Genomics Consortium (PGC)^27^. However, analyses comparing samples from the Schizophrenia Working Group of the PGC found no differences in PRS across consensus DSM/ICD diagnosis (by psychiatrists), diagnostic interview, medical records, and mixed methods^28^. To our knowledge, there is no published research comparing a self-reported clinical diagnosis of schizophrenia from a health professional against a gold-standard research interview diagnosis. In this study, we address this knowledge gap and assess whether a self-reported clinical diagnosis of schizophrenia is a valid approach to identify relevant individuals for genomic research.

## Methods

### Participants

**Fig. 1:**
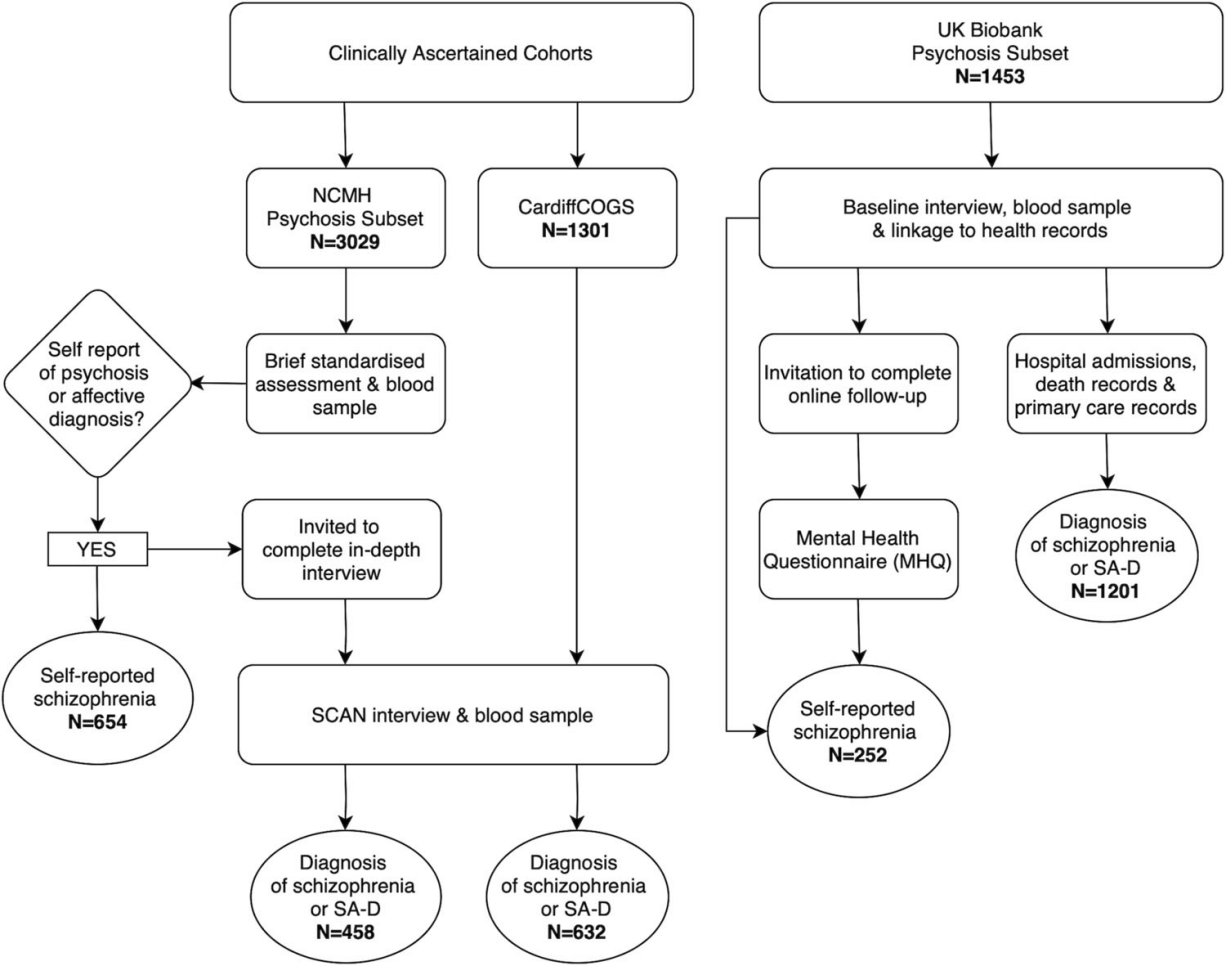
Sample recruitment, assessment methods and number of participants. Flowchart showing the methods used to assess participants in each sample (NCMH, CardiffCOGS and UK Biobank), along with information on the number of participants in each sample, broken down by method of diagnosis. Numbers are provided for schizophrenia. In NCMH, of the 1943 participants who self-reported psychosis: 493 reported schizoaffective disorder, 1001 reported psychosis and 634 reported bipolar disorder. Note that participants could select all diagnoses that they had ever been given so some participants reported more than one psychotic diagnosis.

Study participants came from two clinically-ascertained Cardiff University cohorts, the National Centre for Mental Health (NCMH) and CardiffCOGS, and from the UK Biobank. All participants provided written informed consent. Table 1 provides information on the assessments used to determine diagnosis in each sample. A flowchart of the samples, methods and number of participants recruited is shown in Fig. 1.

**Table 1:** Diagnosis definitions for self-reported, research interview, and medical record diagnosis groups.

Sample	Diagnosis type	Diagnosis subtype	Source	Diagnosis question/variable
**Clinically-ascertained Sample**	Self-report	Lifetime clinical diagnosis	Brief standardized assessment	“Has a doctor or health professional ever told you that you have any of the following diagnoses?”
		Participant opinion	Brief standardized assessment	“What is your primary diagnosis, in your opinion?”
		Current clinical diagnosis	Brief standardized assessment	“What is your primary diagnosis, in your clinician’s opinion?”
	Research interview diagnosis		SCAN-based clinical interview	DSM-IV (1994), DSM-5 (2013) and ICD-10 (1992) diagnoses
**UK Biobank**	Self-report		Mental health questionnaire	“Have you been diagnosed with one or more of the following mental health problems by a professional, even if you don’t have it currently?”
			Initial assessment	Verbal self-report of a mental health diagnosis during interview with a nurse
	Medical record diagnosis		Primary care, hospital admission and death records	ICD-10 diagnosis

The diagnosis type and subtypes by diagnosis source and questions/variables used to derive definitions.

#### NCMH

NCMH participants were recruited via health care services, voluntary organisations or via public advertisement^29^. Trained researchers administered a brief standardized assessment to gather demographic and clinical information and participants were asked to provide a sample for DNA extraction and genetic analyses. Participants self-reporting a schizophrenia, psychosis or affective diagnosis were invited to take part in a research interview based on the Schedules for Clinical Assessment in Neuropsychiatry (SCAN)^30^. NCMH received approval from Health Research Authority and Wales Research Ethics Committee (REC) 2 (16/WA/0323).

#### CardiffCOGS

CardiffCOGS participants were recruited from community, in-patient and voluntary sector mental health services across the UK^31^. All participants completed a SCAN-based research interview^30^, underwent a case-note review and were asked to provide a sample for DNA extraction and genetic analyses. CardiffCOGS received approval from Southeast Wales REC (07/WSE03/110). CardiffCOGS participants were included to increase the sample size for the genetic analysis. These participants were not included in comparisons of self-report and research diagnoses, as self-report diagnoses are not available in this sample.

#### UK Biobank

UK Biobank is a population-based UK cohort of around 500,000 participants, aged between 40–69 at recruitment^32^. Participants completed a range of assessments and provided a sample for genetic analysis. Ethical approval was granted by the Northwest Multi-Centre Ethics Committee. This study was conducted under UK Biobank project number 13310.

### Diagnosis definitions

Table 1 provides an overview of the self-reported, research interview and medical record diagnosis definitions used in this study.

### Self-reported diagnosis

In NCMH, participants were asked whether a doctor or health professional had ever told the participant that they had a mental health diagnosis and prompted with a list of psychiatric diagnoses to choose from (Supplementary Fig. 1). In UK Biobank, participants were asked to report if a doctor had told them they had any serious medical condition in the initial assessment. A subset of participants in the UK Biobank (31%) completed the Mental Health Questionnaire (MHQ), where they were prompted with a list of psychiatric diagnoses to choose from (Supplementary Fig. 2). For both NCMH and UK Biobank, if the participant chose schizophrenia from the list or they verbally self-reported a schizophrenia diagnosis, they were assigned a schizophrenia self-reported diagnosis in this study. Table 1 describes the subtypes of self-reported diagnoses available in the clinically-ascertained sample. A self-reported schizoaffective disorder diagnosis was excluded from analyses, as it was not possible to differentiate between the depressed and manic subtypes.

### Research interview diagnosis

In the clinically-ascertained samples (NCMH and CardiffCOGS), DSM-IV, DSM-5, and ICD-10 research diagnoses were derived from a SCAN-based clinical interview and note review where available. A research interview diagnosis of schizophrenia was given in this study if either a DSM or ICD schizophrenia criteria were met. If participants met criteria for schizoaffective disorder depressed-type (SA-D), they were also included alongside participants with schizophrenia given evidence that these participants do not differ on a range of phenotypic and genotype measures, including symptoms, cognition and polygenic risk^33^. ‘Other psychotic disorders’ in this study refer to the following diagnoses: psychosis not otherwise specified, schizophreniform disorder, delusional disorder, brief psychotic disorder, acute polymorphic disorder, and other psychotic illness.

### Medical record diagnosis

In UK Biobank, a medical record diagnosis of schizophrenia and SA-D were defined as a F20/F25.1 ICD-10 code from national hospital admission records or death records, or an equivalent read code from primary care (Supplementary Table 1). Hospital records date back to 1997 for England, 1998 for Wales and 1981 for Scotland and contain coded data on admissions, operations, and procedures. Primary care data was obtained for approximately 45% of the UK Biobank cohort. In secondary analyses, hospital admissions for schizophrenia were further subdivided into primary and secondary admissions. Primary ICD-10 codes represent conditions that caused the admission and secondary ICD-10 codes represent conditions that coexist at the time of admission, affect the treatment received, or develop after admission.

### Unaffected controls

Unaffected controls for the clinically-ascertained samples were NCMH participants with no history of a mental health diagnosis and who were recruited through participants with a psychiatric diagnosis (e.g., a family member/partner) or via advertisements. Unaffected controls for the UK Biobank analyses consisted of participants in UK Biobank who did not have a psychotic disorder diagnosis (F21-F29 inclusive) from admission records, death records, primary care records, or from self-reported sources.

### Phenotypic data

The phenotypes compared across diagnostic groups included sex, age at interview (in years), educational attainment, and employment status. Educational attainment was dichotomised to GCSE (General Certificate of Secondary Education) and above, usually achieved at 16 years upon completing high school, or below GCSE/no qualification, consistent with previous research^34^, in addition to degree/no degree. Employment status was dichotomised to in current paid employment or not and restricted to participants under the age of 65 who did not report being retired.

### Genetic data

#### Clinically-ascertained sample

The clinically-ascertained participants were genotyped on the Illumina OmniExpress (Infinium OmniExpress-24 Kit), Illumina PsychArray (Infinium PsychArray-24 Kit) or Illumina GSA (Infinium Global Screening Array-24 Kit) genotyping platforms. Quality control and imputation using the Haplotype Reference Consortium (HRC)^35^ was performed as part of the DRAGON-Data protocol^36^. Datasets containing participants from the clinically-ascertained samples were restricted to those with the diagnoses described above and who did not carry a neurodevelopmental CNV^36^. These samples were combined with samples from 1000 Genomes European phase 3^37^ using PLINK v1.9^38^ after restricting to overlapping SNPs. The 1000 Genomes sample was included to provide a population reference to allow studies using different arrays to be directly compared^39^. The following quality control exclusion criteria were subsequently applied to SNPs: minor allele frequency (MAF) < 0.05, genotyping rate < 0.05, and Hardy-Weinberg equilibrium *p* ≤ 10^−6^. Linkage disequilibrium-pruned SNPs (500 variant count window size, 20 variant count to shift the window at the end of each step, a pairwise r^2^ threshold of 0.2) were used to identify related individuals and to derive principal components (PC). One individual from each pair assumed to be duplicates (kinship coefficient > 0.98) or related (kinship coefficient > 0.1875) was removed at random. The first 5 PCs were used to perform multi-dimensional clustering to identify an ancestrally-homogenous subsample of individuals^40^. The first 5 PCs explained the majority of the variance in the principal components, adding additional PCs did not change the classifications. Individuals within a 90% threshold from the most central point were included for analyses. There were insufficient numbers of participants of non-European ancestries in NCMH and CardiffCOGS to allow us to analyse PRS in different ancestries.

#### UK Biobank

Imputed genetic data were provided by UK Biobank. Pre-imputation quality control and imputation have been described elsewhere^41^. Briefly, participants were assayed at the Affymetrix Research Services laboratory using the UK Biobank Axiom or UK BiLEVE Axiom purpose-built arrays. Imputation was completed using the HRC panel^35^. We applied additional quality control procedures using the same thresholds used in our clinically-ascertained sample and detailed elsewhere^39,42^. Genetic analyses were restricted to participants with European ancestry, to mirror the clinically-ascertained sample, using the method described above, see also Legge et al^42^.

### Polygenic risk scores

In the clinically-ascertained sample and UK Biobank, PRSicev2^43^ was used to calculate PRS for schizophrenia using GWAS de-duplicated summary statistics that were derived separately from our clinical sample and UK Biobank^28^. PRS were also calculated for bipolar disorder^13^ and major depressive disorder^44^. Summary statistics underwent quality control^36^ and SNPs with MAF > 0.01 outside of the major histocompatibility complex region were used in the PRS analysis. PRS were calculated, using relatively independent SNPs (r^2^ < 0.1, within 500 kb window), at a *p*-value threshold of 0.05^28^. Polygenic risk scores were standardised within samples prior to analysis.

### Analysis

In NCMH, positive predictive values (PPV) were used to assess the ratio of participants with a self-reported schizophrenia diagnosis from a health professional who had a concordant DSM/ICD research interview diagnosis. We also considered a research interview diagnosis of schizophrenia and schizoaffective disorder depressive-type (SA-D) together as there is evidence these two groups do not substantially differ with respect to genetic liability to schizophrenia^28,33^. It was not possible to assess negative predictive values (NPV), sensitivity and specificity in the clinically-ascertained sample due to the recruitment methods; participants were only approached to complete a SCAN-based research interview if they self-reported a mood or psychotic disorder diagnosis.

In the UK Biobank, PPV, NPV, sensitivity and specificity were used to assess how predictive a self-reported clinical diagnosis from a health professional was of a medical record diagnosis. We scaled the PPV and NPV to the population point prevalence of schizophrenia (0.6%) (Supplementary Note 1). We could not calculate PPV related to a medical record diagnosis of schizophrenia and SA-D together due to a very low prevalence of SA-D in the UK Biobank.

In both NCMH and the UK Biobank, logistic regressions were used to test for phenotypic differences between individuals that only self-reported a diagnosis and those who had a research interview diagnosis/medical record diagnosis (some of whom also self-reported). Year of birth and sex were included as covariates.

Due to the limited number of genotyped participants in NCMH, the genetic analyses included participants from both NCMH and CardiffCOGS. In both the clinically-ascertained sample and the UK Biobank logistic regressions were used to test for genetic differences in schizophrenia between self-report-only and the research interview diagnosis/medical record diagnosis groups.

We compared the variance explained by schizophrenia PRS on the liability-scale (r^2^, assuming 1% lifetime risk) in schizophrenia case/control status in the clinically-ascertained sample and UK Biobank, separated by diagnosis definitions, against the variances reported by other samples of European genetic ancestry in the PGC3 schizophrenia GWAS. The r^2^ values refer to the variance explained by the schizophrenia PRS in comparison to a covariates-only baseline model. In addition, we calculated the variance explained in schizophrenia case/control status in UK Biobank for bipolar disorder^13^ and major depressive disorder^44^ PRS.

In the UK Biobank sample, further logistic regressions were used to assess if schizophrenia PRS was associated with the number of times a diagnosis was reported, the number of admissions and type of admission (primary and secondary). These PRS analyses were covaried for the first 5 PCs, array, age at assessment, and sex.

All statistical tests were two-sided. Unless otherwise specified, data analysis was conducted in R.

## Results

We identified participants with a self-reported, research or medical record diagnosis of either schizophrenia or schizoaffective disorder (SA-D) from across the three samples. This included 1112 participants from NCMH, 632 participants from CardiffCOGS and 1453 participants from the UK Biobank. Unaffected controls included 749 participants from NCMH and 501,088 participants from UK Biobank. Demographic information for each sample is provided in Table 2. The number of participants included in each analysis varies according to the availability of data (e.g. number with both self-report and research/medical record diagnoses for PPI analyses and number of participants with genotype data) and are detailed in the relevant sections.

**Table 2:** Sample characteristics by cohort.

	*N*	Male *n* (%)	Mean age (SD)	High school educated *n* (%)	Degree *n* (%)	Employed *n* (%)*	Ethnicity				
							White	Black	Asian	Mixed Race	Other
**Self-report only of schizophrenia**											
NCMH	654	417/654 (64%)	46.58 (13.18)	362/498 (73%)	80/498 (16%)	59/490 (12%)	557/643 (87%)	22/643 (3%)	33/643 (5%)	20/643 (3%)	11/643 (2%)
UK Biobank	252	156/252 (62%)	55.33 (7.94)	174/242 (72%)	86/242 (36%)	58/226 (26%)	224/252 (88.9%)	11/252 (4.4%)	6/252 (2.4%)	5/252 (2.0%)	6/252 (2.4%)
**Research diagnosis of schizophrenia or SA-D**											
NCMH	458	287/458 (63%)	43.42 (12.97)	254/311 (82%)	56/311 (18%)	41/270 (15%)	408/445 (92%)	7/445 (2%)	12/445 (3%)	12/445 (3%)	6/445 (1%)
CardiffCOGS	632	416 (66%)	43 (12)	383/618 (62%)	73/618 (12%)	58/606 (10%)	621/631 (98%)	<5/631 (<1%)	<5/631 (<1%)	5/631 (<1%)	0/631 (0%)
**Medical record diagnosis of schizophrenia or SA-D**											
UK Biobank	1201	735/1201 (61%)	54.58 (8.40)	715/1103 (65%)	273/1103 (25%)	177/1057 (17%)	984/1149 (85.6%)	78/1149 (6.8%)	36/1149 (3.1%)	27/1149 (2.3%)	24/1149 (2.1%)
**Unaffected controls**											
NCMH	749	276 (37%)	50 (19)	564/612 (92%)	298/612 (49%)	308/565 (55%)	714/744 (96%)	5/744 (<1%)	13/744 (1.7%)	9/744 (1.2%)	3/744 (<1%)
UK Biobank	501,088	228,245 (46%)	57 (8)	347,702/465,252 (75%)	160,819/ 465,252 (35%)	278,663/ 427,382 (65%)	470,020/ 494,947 (95%)	7937/ 494,947 (1.6%)	8011/ 494,947 (1.6%)	2904/ 494,947 (0.6%)	6075/ 494,947 (1.2%)

Columns refer to NCMH, CardiffCOGS and UK Biobank participants with or without (controls) schizophrenia or schizoaffective disorder, depressed type (SA-D), number of participants, number and percentage (%) of male sex, educated and employed (*filtered to under 65 years), mean age in years at recruitment interview and standard deviation (SD).

### Positive predictive values

In NCMH, the proportion of participants with a self-reported schizophrenia diagnosis from a health professional who had a research interview diagnosis was used to estimate PPV (*n* = 273, Table 3). A self-reported current clinical diagnosis of schizophrenia had a PPV of 0.74 for receiving a research interview diagnosis of schizophrenia, 0.85 of receiving a diagnosis of schizophrenia or SA-D and 0.9 of receiving a diagnosis of schizophrenia, SA-D or any other psychotic disorder. A self-reported current clinical diagnosis produced slightly higher PPVs than self-reported lifetime clinical diagnoses or participant opinion diagnoses (Table 3). Secondary PPV analyses indicated that a self-reported psychotic disorder was less predictive of receiving a research diagnosis of schizophrenia, SA-D or other psychotic disorder (PPVs range: 0.27–0.65, Supplementary Table 2). All participants who self-reported schizophrenia but did not proceed to get a research interview diagnosis of schizophrenia received other mood or psychotic research diagnoses, except one participant where there was insufficient data to make a research interview diagnosis (Supplementary Table 3).

**Table 3:** Positive predictive values for self-reported diagnoses of schizophrenia and subsequent research interview diagnoses.

Self-report method	Research interview diagnosis	Total number of participants	SZ self-report & SZ* research diagnosis	SZ self-report & non-SZ research diagnosis	PPV
Lifetime clinical diagnosis	Schizophrenia	273	190	83	0.70
	Schizophrenia/SA-D		222	51	0.81
	Schizophrenia/SA-D/other psychotic disorders		238	35	0.87
Current clinical diagnosis	Schizophrenia	239	176	63	0.74
	Schizophrenia/SA-D		202	37	0.85
	Schizophrenia/SA-D/other psychotic disorders		215	24	0.90
Participant opinion	Schizophrenia	102	77	25	0.75
	Schizophrenia/SA-D		84	18	0.82
	Schizophrenia/SA-D/other psychotic disorders		87	15	0.85

Positive predictive values for self-reported schizophrenia diagnoses in NCMH. Columns represent the self-reported method, the research interview diagnoses, the total number of participants, and the number of individuals who had a (i) schizophrenia self-reported and subsequent schizophrenia (plus SA-D/other psychotic disorders) research diagnosis, (ii) schizophrenia self-report and non-schizophrenia research diagnosis and PPV.

*PPV* positive predictive value, *SZ* schizophrenia, *SA-D* schizoaffective disorder depressive-type. * Schizophrenia, schizophrenia/SA-D, and Schizophrenia/SA-D/other psychotic disorders combinations tested.

In UK Biobank, predictive values were calculated in participants who had *both* a self-reported diagnosis and a medical record diagnosis. After correction for the point prevalence^45^ of schizophrenia (0.6%), the PPV of having a medical record diagnosis of schizophrenia for those who self-reported a schizophrenia diagnosis was 0.83, the NPV 0.996, the specificity 0.9995 and the sensitivity 0.383 (Table 4). When including a medical record diagnosis of schizophrenia or any other psychotic-related disorder the specificity decreased to 0.77 and sensitivity to 0.21 (Supplementary Table 4).

**Table 4:** Predictive values for self-reported schizophrenia and medical record diagnosis of schizophrenia.

		Medical record diagnosis of schizophrenia		
		**Yes**	**No**	
**Self-reported diagnosis of schizophrenia**	**Yes**	450	156	PPV*: 0.853
	**No**	724	333784	NPV*: 0.996
		Sensitivity: 0.383	Specificity: 0.9995	

Positive predictive values (PPV), negative predictive values (NPV), sensitivity and specificity of individuals who self-reported schizophrenia either verbally to a nurse on the initial assessment or on the mental health questionnaire and had a medical record diagnosis of schizophrenia. *Adjusted values based on point prevalence (unadjusted PPV = 0.7425743, unadjusted NPV = 0.9978356).

### Phenotypic and genetic differences across diagnosis source

#### Clinically-ascertained sample

**Fig. 2:**
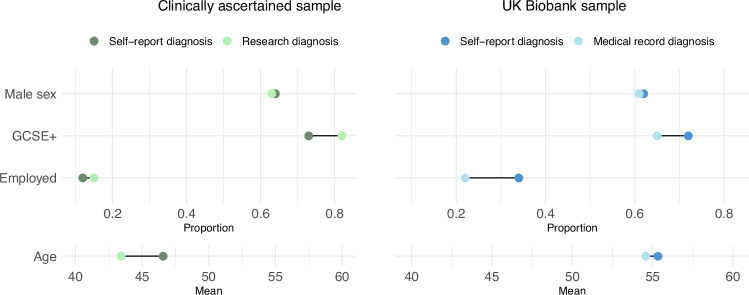
Phenotype differences across methods of diagnosis. Cleveland plot of the proportion of participants that were male, had a GCSE qualification or above, were employed, and mean age by those who had a self-report diagnosis only and a research interview diagnosis/medical record diagnosis.

We compared 458 participants in NCMH with a research interview diagnosis of schizophrenia or SA-D with 654 participants whose only source of diagnosis was a self-reported diagnosis of schizophrenia. Participants who had a research interview diagnosis were younger (mean age 43 vs. 47; OR = 0.77; 95% CI = 0.67, 0.88; *p* = 9.11 × 10^-5^) and more likely to have a high school qualification (GCSE) or above (OR = 1.61; 95% CI = 1.13, 2.29; *p* = 0.008) than self-reporting only participants. Having a degree did not significantly differ across self-report only and research interview groups (OR = 1.15, 95%CI = 0.79, 1.67, *p* = 0.47). No significant differences were detected in employment (OR = 1.35; 95% CI = 0.87, 2.08; *p* = 0.18) or sex (OR = 1.07; 95% CI = 0.83, 1.37; *p* = 0.61) (Fig. 2).

**Fig. 3:**
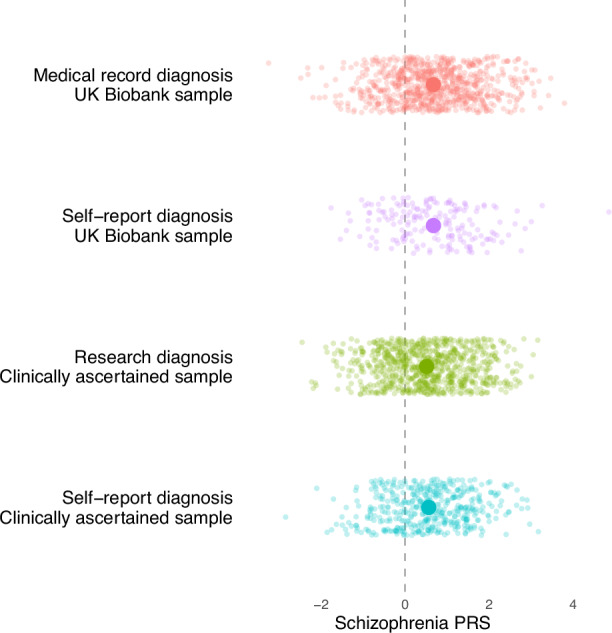
Schizophrenia polygenic risk scores plotted by method of diagnosis. Standardised polygenic risk scores for each method of diagnosis across both samples. The bold circles represent the mean of each diagnostic group. PRS; polygenic risk score.

For the genetic analyses, we added participants from the CardiffCOGS cohort (who all had a research diagnosis) to the NCMH sample to increase the sample size. Therefore, the genetic analyses included 803 participants with a research diagnosis, 449 participants who exclusively had a self-report diagnosis and 710 controls. We found no significant difference in schizophrenia PRS between participants who had a research interview diagnosis and those who only self-reported a diagnosis (OR = 0.97; 95% CI = 0.86, 1.09; *p* = 0.59) (Fig. 3).

CardiffCOGS samples were genotyped on a different array platform (OmniExpress) to NCMH cases and controls, which were split across GSA and PsychChip (Supplementary Table 5). To test whether there were any batch effects, we removed the CardiffCOGS samples from the case/control analysis. We found a consistent effect for the association between PRS and schizophrenia case/control status (OR = 1.70; 95%CI = 1.44-2.02; *P* = 1.91 × 10^−10^, r^2^ = 0.036; se = 0.011; AUC = 0.64). This finding, alongside Supplementary Fig. 3 suggests that there were no batch effects in the genetic data.

#### UK Biobank sample

Compared to participants whose basis for a diagnosis was solely self-report (*n* = 252), participants who had a medical record diagnosis of schizophrenia or SA-D (*n* = 1201) were less likely to be in paid employment (OR = 0.55; 95% CI = 0.39, 0.79; *p* = 0.001), and less likely to have a GCSE (high school) or higher qualification (OR = 0.70; 95% CI = 0.51, 0.95; *p* = 0.02). Furthermore, participants with a medical record diagnosis were less likely to have a degree (OR = 0.59, 95%CI = 0.44, 0.79, *p* = 0.0005). There were no differences in sex (OR = 0.95; 95% CI = 0.72, 1.26; *p* = 0.75) or age across the groups (OR = 0.91; 95% CI = 0.80, 1.04; *p* = 0.18) (Fig. 2).

The genetic analysis included 181 participants with only a self-report and 809 participants with a medical record diagnosis. No significant difference in schizophrenia PRS was found between participants who had a medical record diagnosis and a self-report diagnosis (OR = 1.01; 95%CI = 0.87,1.19; *p* = 0.85; Fig. 3).

#### Liability explained in case/control status

**Fig. 4:**
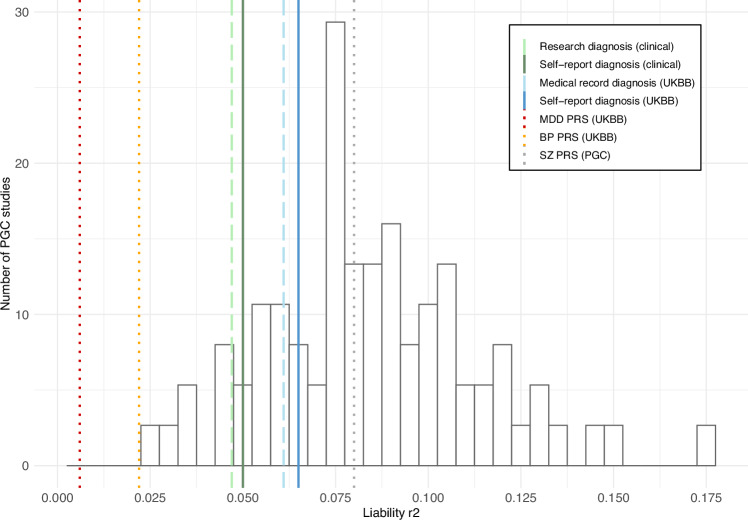
Variance explained by schizophrenia PRS by diagnostic method compared to PGC studies. The variance explained by schizophrenia PRS in schizophrenia case/control status on the liability scale assuming a 1% population prevalence of European genetic ancestry PGC cohorts. The lines plotted on the graph represent the r^2^ of each diagnostic group. SZ, MDD and BP PRS are plotted as a reference. Clinical clinically-ascertained sample, UKBB UK Biobank, MDD major depressive disorder, BP bipolar disorder, SZ schizophrenia, PGC Psychiatric Genomics Consortium.

The proportion of variance on the liability scale attributable to schizophrenia PRS in both diagnostic groups in the clinically-ascertained sample and UK Biobank studies fell within the distribution of studies in the latest PGC analysis (Fig. 4, Table 5). In the clinically-ascertained sample, the schizophrenia PRS explained 5.0% of the variability in the self-reported-only group, and 4.7% in the research interview diagnosis group. In the UK Biobank sample, the schizophrenia PRS explained 6.5% of the variability in the self-reported only-group and 6.1% in the medical record diagnosis group (Table 5).

**Table 5:** Variance explained by schizophrenia PRS for each diagnostic method.

	Case/control	Definition	Number of participants	OR	95% CI	*P*	R^2^	se	AUC
***Clinical sample***									
Self-report only	Case	Self-reported schizophrenia	552	1.89	1.67–2.15	7.33 × 10^-26^	0.050	0.007	0.67
	Control	Unaffected controls	710						
Research interview	Case	Research interview diagnosis schizophrenia/SA-D	789	1.83	1.64–2.05	1.02 × 10^-28^	0.047	0.007	0.66
	Control	Unaffected controls	710						
***UK Biobank sample***									
Self-report only	Case	Self-reported schizophrenia	494	2.01	1.84–2.20	6.18 × 10^-53^	0.065	0.009	0.69
	Control	No schizophrenia or psychotic disorder diagnosis (F20:F29) in first occurrences field (ID = 2405)	401795						
Medical record diagnosis	Case	Medical record schizophrenia/SA-D (F20/F25.1)	809	1.96	1.83–2.11	3.35 × 10^-80^	0.061	0.006	0.68
	Control	No schizophrenia or psychotic disorder diagnosis (F20:F29) in first occurrences field (ID = 2405)	401841						
***Other UK Biobank PRS***									
Bipolar PRS	Case	Any schizophrenia diagnosis	990	1.50	1.41–1.60	6.88 × 10^-37^	0.022	0.003	0.61
	Control	No schizophrenia, psychotic or mood disorder diagnosis (F20:F39) in first occurrences field (ID = 2405)	352972						
Depression PRS	Case	Any schizophrenia diagnosis	990	1.23	1.15–1.30	1.91 × 10^-10^	0.006	0.002	0.56
	Control	No schizophrenia, psychotic or mood disorder diagnosis (F20:F39) in first occurrences field (ID = 2405)	352972						

The proportion of variance on the liability scale attributable to schizophrenia PRS in those with a self-reported diagnosis and a SCAN-based research interview diagnosis in the clinically ascertained sample and self-reported diagnosis and medical record diagnosis in UK Biobank. Additionally, Bipolar PRS and Depression PRS in the UK Biobank explaining schizophrenia case/control status are provided as a reference. Multidimensional clustering was completed in both cases and controls for each of these analyses. *SA-D* schizoaffective disorder depressive-type, *OR* Odds ratio, *95%CI* 95% confidence intervals, *P* *p*-value, *r2* variance explained by PRS, *se* standard error, *AUC* area under the curve.

### Further examination of diagnosis source in the UK Biobank

Schizophrenia PRS increased with the number of times a schizophrenia diagnosis was reported; OR = 1.82 (95%CI = 1.67, 1.99) for 1 endorsement compared to controls and OR = 2.11 (95%CI = 1.92, 2.32) for 2 or more endorsements (Supplementary Fig. 4). Participants who had two or more diagnosis endorsements had a significantly higher schizophrenia PRS than participants who only had one diagnosis endorsement (OR = 1.15; 95%CI = 1.01, 1.31; *P* = 0.03). The schizophrenia PRS also increased as the number of schizophrenia hospital admissions increased from OR = 1.85 (95%CI = 1.72, 2.00) for 0 admissions (participants had an alternative source of schizophrenia diagnosis), to OR = 1.92 (95%CI = 1.77, 2.08) for 1 admission, and OR = 2.28 (95%CI = 2.01, 2.58) for 2 or more admissions (Supplementary Fig. 5).

Schizophrenia cases with a primary ICD-10 admission code had a higher schizophrenia PRS than those who had schizophrenia as a secondary ICD-10 admission code (OR = 1.28; 95%CI = 1.10, 1.49; *P* = 0.002). Participants identified with a schizophrenia diagnosis from a secondary code only, on average, had lower schizophrenia PRS than those identified from self-reported or a primary hospital admission code (Supplementary Fig. 6). These findings did not appear to be related to the secondary code only participants having different associated diagnoses (Supplementary Table 6 and 7).

## Discussion

In this study, we demonstrated that participants who self-reported a clinical diagnosis of schizophrenia were likely to be given a subsequent research interview diagnosis of schizophrenia, SA-D or other psychotic disorder (PPVs between 0.70 and 0.90). Furthermore, we found that participants in UK Biobank who self-reported a clinical schizophrenia diagnosis were likely to have a medical record diagnosis of schizophrenia or another psychotic disorder (PPV = 0.80). Although we found some phenotypic differences, genetic liability to schizophrenia did not significantly differ between participants with a self-reported diagnosis compared to those diagnosed via research interview or medical records. The variance explained by the schizophrenia PRS for all diagnostic methods fell within the distribution of PGC studies. These findings suggest that using a self-reported clinical diagnosis of schizophrenia is a valid approach for identifying participants for large-scale genomic research.

In the clinically-ascertained sample, participants who self-reported schizophrenia were likely to receive a research diagnosis of schizophrenia, SA-D or other psychotic disorder, however, participants who self-reported a lifetime clinical diagnosis of psychosis (without schizophrenia and bipolar) were much less likely to obtain a research interview diagnosis of schizophrenia, SA-D, or other psychotic disorder (PPVs 0.27–0.65). Previous research has shown that a schizophrenia diagnosis has much better agreement between diagnostic methods (PPVs 0.69-1.00) than other diagnoses such as bipolar, depression and other psychotic disorders^9^. Although self-reported diagnoses have generally been shown to have poor predictive accuracy when it comes to obtaining a gold-standard research interview diagnosis^46,47^, our results suggest that for schizophrenia specifically, self-reported diagnoses could be used in place of a research interview diagnosis to identify participants in genomic research. No research diagnostic method, including clinical interviews, is totally free of bias and recruiting participants via a range of sources is likely to lead to more representative studies as a whole.

In UK Biobank, participants who self-reported schizophrenia were likely to have a medical record diagnosis of schizophrenia. However, the low sensitivity values indicate that a self-report in the UK Biobank did not capture everyone who had a medical record. This could be for many different reasons including later onset of illness, the stigma associated with reporting a schizophrenia diagnosis, or the non-specific nature of the question in the initial assessment (“Has a doctor ever told you that you have any other serious medical conditions?”). Participants were not specifically asked about mental health and prompts, if any, were only given for physical health conditions. This may have led to under-reporting of schizophrenia and underscores the importance of ensuring self-report questions specifically reference the diagnosis of interest. The high negative classifications in UK Biobank illustrated that participants who did not self-report schizophrenia also did not have a medical record of schizophrenia and vice versa, demonstrating that, although certainly enhanced by the low prevalence, self-reported diagnoses are effective at ruling out non-cases.

Currently, using a research interview and note review to obtain a diagnosis is considered gold standard, although we find the requirement to attend and undergo a detailed and time-consuming interview may induce recruitment biases, with those participating in such an interview (after the majority having a brief interview first) being younger and more likely to have a high school qualification (GCSEs) than those who only have self-reported. No difference in degree qualification was observed across groups, however, this is likely due to the small proportion of individuals with a degree in the clinical sample (research interview = 18% vs self-report = 16%). Research interviews may exclude participants who are more acutely unwell or cognitively impaired and unable to complete a long assessment. In the UK Biobank, participants with a medical record diagnosis were less likely to have GCSEs and to be employed. Furthermore, participants with a medical record diagnosis were less likely to have a degree (medical record = 25% vs self-report = 36%). This suggests that these participants may have more impaired functioning than those with a self-report only, and by using medical records only as researchers we may be missing participants who are functioning well and/or have not been admitted to hospital. An alternative explanation is that those who did not self-report schizophrenia in UK Biobank may not have sufficient insight to verbally report their diagnosis to the research nurse or may not have been able to complete the online Mental Health Questionnaire due to poorer functioning or education. Thus, the self-report sample in UK Biobank may be missing participants with more impaired functioning. Taken together, our results highlight some phenotypic differences between different methods of identifying a diagnosis of schizophrenia, particularly within UK Biobank where sample representativeness is a known issue. However, for clinically ascertained samples, our results suggest that self-report of a diagnosis made by a health professional may be sufficient given the limited phenotypic differences between self-report and research interview diagnosis, and the potential to include more participants with lower education using self-report.

In contrast to a depression study in UK Biobank which found participants defined by minimal phenotyping (self-report, help-seeking, and symptom-based) had lower SNP-derived heritability than the strictly defined participants (Composite International Diagnostic Interview)^23^, we did not find a difference in schizophrenia PRS between the self-report and research interview/medical record diagnosis groups. This may reflect differences in obtaining clinical diagnoses of schizophrenia and depression. Depression can be diagnosed in many settings including primary care, whilst schizophrenia is almost universally diagnosed in secondary care following comprehensive assessment. There may be hesitancy to disclose a diagnosis of schizophrenia to patients due to the associated stigma, as there is evidence that health professionals are more likely to communicate diagnoses of depression than schizophrenia to patients, are more likely to diagnose schizophrenia after recurrent episodes than during the first episode and show a preference for using alternative terms to schizophrenia such as psychosis^48–50^. As such, a self-reported diagnosis of schizophrenia may have higher validity.

Our results highlight the potential, especially for genomic studies, of using this self-report method to identify participants for schizophrenia research. However, we did find differences in schizophrenia PRS within hospital admission diagnoses (in primary and secondary admissions). We also found the number of diagnosis reports and admissions were associated with higher PRSs in UK Biobank, as has been reported in previous literature^51,52^. This is consistent with findings from the PGC, who reported schizophrenia PRS to be higher in patients who were recruited from inpatient settings^28^. These findings could indicate greater severity or improved accuracy of diagnosis, or both. We also found participants whose primary reason for admission was schizophrenia, and those who only self-reported schizophrenia, had a higher schizophrenia PRS than those with a secondary admission diagnosis. One explanation of the difference in schizophrenia PRS could be that the secondary admission group were participants who were not admitted primarily for psychosis because their symptoms were milder or were well treated. Alternatively, the accuracy of a secondary diagnosis may be more prone to error than a diagnosis given for a primary admission to hospital (e.g., if admitted for a heart attack), although this did not appear to be the case when looking specifically at psychiatric comorbidities.

### Limitations

It is important to note limitations of the current study. Participants were invited to complete a SCAN-based research interview if they self-reported psychosis or a schizophrenia diagnosis. This study design prevented us from assessing other metrics (negative predictive value, sensitivity, and specificity) in the clinically-ascertained sample. This also meant we were unable to adjust the PPV to the population point prevalence of schizophrenia. As a result, the PPV could have been inflated by the high proportion of schizophrenia participants in our clinically-ascertained sample. Additionally, some participants had their diagnosis confirmed by a clinician if systematically recruited, which could have increased the positive predictive values, and only a subset of the sample were asked their own opinion of their diagnosis (*n* = 99). Despite these limitations, our clinically-ascertained sample is one of the only psychosis-based samples with both self-report diagnosis data and a gold-standard research diagnosis. In the UK Biobank, 93% of the participants with a medical record diagnosis of schizophrenia have a hospital admission, therefore the predictive values primarily reflect how predictive a self-report was of a hospital admission.

Both the clinically-ascertained sample and UK Biobank were sampled from the UK; therefore, the findings may not apply to other countries. The generalisability of the UK Biobank findings are also hindered because this sample is not wholly representative of the UK population^53^. Furthermore, the primary and secondary admission diagnosis in the UK Biobank may have differed depending on the location of the admission (e.g., psychiatric hospital vs general) or by the clinician’s expertise. Polygenic risk scores were restricted to participants from a European genetic ancestry. We were unable to investigate whether the polygenic risk scores differ across diagnostic groups in non-European genetic ancestries due to a limited number of participants in our samples from non-European genetic ancestries. Lastly, the recruitment methods in the NCMH study could have enriched for relatives of those with mood and psychotic disorders, although only 5% (*n* = 33) of our controls reported having a family history of bipolar disorder or schizophrenia. Nonetheless, and although the effect of this would be conservative, this could weaken the variance explained in our schizophrenia PRS analyses. Our findings apply to large-scale genomic studies and caution should be applied in applying self-report methods of diagnosis to studies such as clinical trials where diagnostic accuracy for individual participants is paramount and where participants may receive financial reimbursement for their time.

### Conclusion

Self-reporting a clinical schizophrenia diagnosis may be a valid method for identifying cases in schizophrenia genomic research, providing systematic differences of methodologies are transparently noted. Participants who only self-reported a schizophrenia diagnosis showed differences in age, education, and employment but crucially, they did not differ in relation to schizophrenia genetic liability. These findings provide preliminary evidence for using less stringent methods of ascertaining diagnoses in schizophrenia genomic research, which could reduce the burden on participants and researchers to complete extensive interviews, and thereby potentially improve the representativeness of future samples and increase sample sizes.

## Supplementary information

Supplementary Materials

## Data Availability

UK Biobank data can be obtained upon application from https://www.ukbiobank.ac.uk/enable-your-research. The de-duplicated PGC summary stats for schizophrenia are available on the PGC website https://figshare.com/articles/dataset/scz2022/19426775. All code will be made available upon request. Data from the clinically-ascertained cohorts cannot be made publicly available due to restrictions in ethical approvals.
