# Supplementary material for: Assessing the validity of a self-reported clinical diagnosis of schizophrenia
Source: Schizophrenia (Heidelb). 2024 Oct 30;10(1):99. doi: 10.1038/s41537-024-00526-5 (PMC11526013; doi:10.1038/s41537-024-00526-5)
Supplement: Supplementary file 1 — Supplementary Materials [file 41537_2024_526_MOESM1_ESM.pdf]

Supplementary Materials

Assessing the validity of a self-reported clinical diagnosis of schizophrenia.

Grace E Woolway; Sophie E Legge; Amy Lynham; Sophie E Smart; Leon Hubbard; Ellie R Daniel; Antonio F Pardiñas; Valentina Escott-Price; Michael C O'Donovan; Michael J Owen; Ian R Jones; James TR Walters.

Table of Contents

Supplementary Table 1: UK Biobank schizophrenia diagnosis definition 2

Supplementary Table 2: PPVs for psychosis in the clinically-ascertained sample 2

Supplementary Table 3: Diagnoses of participants who did not receive a schizophrenia or SA-D research diagnosis 3

Supplementary Table 4. Predictive values for self-reported schizophrenia and medical record diagnosis of schizophrenia or other psychotic disorders. 3

Supplementary Table 5: Diagnosis variables used for self-reported, research interview, and medical record diagnosis groups. 4

Supplementary Table 6: Number of other psychotic and mood admissions by primary and secondary schizophrenia admission groups 4

Supplementary Table 7: Other admission diagnoses by primary and secondary schizophrenia admission groups 4

Supplementary Figure 1: NCMH self-reported diagnoses 5

Supplementary Figure 2: Self-reported diagnoses in UK Biobank's mental health questionnaire 6

Supplementary Figure 3: Principal components 1-6 by array 7

Supplementary Figure 4: Diagnosis endorsements and schizophrenia PRS 8

Supplementary Figure 5: Hospital admissions for schizophrenia and schizophrenia PRS 8

Supplementary Figure 6: Schizophrenia PRS and primary and secondary admission groups 9

Supplementary Note 1: PPV/NPV prevalence adjustment 9

Supplementary Table 1: UK Biobank schizophrenia diagnosis definition

UK Biobank schizophrenia diagnosis	Field ID
Medical record diagnosis	
Primary care diagnosis	130875 (codes 30/31)
Hospital admission	130875/130885 (codes 40/41)
- Primary hospital admission	41202
- Secondary hospital admission	41204
Death records	130875 (codes 20/21)
Self-report diagnosis	
Mental health questionnaire	20544 (code 2)
Verbal self-report	20002 (code 1289)

The UK Biobank field identifiers used to create the schizophrenia diagnosis in UK Biobank.

Supplementary Table 2: PPVs for psychosis in the clinically-ascertained sample

Self-report method	Research diagnosis	Total number of participants who self-report psychosis (without schizophrenia/bipolar)	PSY self-report & SZ* research diagnosis	PSY self-report & non-SZ research diagnosis	PPV
**Lifetime clinical diagnosis**	Schizophrenia	117	33	84	0.28
	Schizophrenia/SA-D		58	59	0.50
	Schizophrenia/SA-D/other psychotic disorders		71	46	0.61
**Current clinical diagnosis**	Schizophrenia	94	25	69	0.27
	Schizophrenia/SA-D		45	49	0.48
	Schizophrenia/SA-D/other psychotic disorders		58	36	0.62
**Participant opinion**	Schizophrenia	51	22	29	0.43
	Schizophrenia/SA-D		27	24	0.53
	Schizophrenia/SA-D/other psychotic disorders		33	18	0.65

Positive predictive values (PPV) for individuals who self-reported psychosis (without also reporting schizophrenia or bipolar disorder) in NCMH. Columns represent the self-reported method, the research interview diagnoses, total number of participants, and the number of individuals who had a (i) psychosis (- bipolar/schizophrenia) self-report and subsequent schizophrenia (plus SA-D/other psychotic disorders) research diagnosis, (ii) psychosis (- bipolar/schizophrenia) self-report and non-schizophrenia research diagnosis, and PPV. PPV, positive predictive value; SZ, schizophrenia; SA-D, schizoaffective disorder depressive-type. * Schizophrenia, schizophrenia/SA-D, and Schizophrenia/SA-D/other psychotic disorders combinations tested.

Supplementary Table 3: Diagnoses of participants who did not receive a schizophrenia or SA-D research diagnosis

DSM 4 Diagnosis	Number of participants
Alcohol induced psychosis	2
Bipolar disorder type 1	8
Brief psychotic disorder	2
Cyclothymia	2
Delusional disorder	1
Major depressive disorder recurrent	5
Major depressive disorder single episode	1
Psychosis not otherwise specified	8
Psychotic depression	4
Schizoaffective bipolar type	15
Substance induced psychotic disorder	2
Unknown	1

DSM 4 diagnoses of participants who self-reported schizophrenia and did not receive a SCAN-based research interview diagnosis of schizophrenia or schizoaffective disorder depressive type (SA-D) (n=51).

Supplementary Table 4. Predictive values for self-reported schizophrenia and medical record diagnosis of schizophrenia or other psychotic disorders.

	**Medical record schizophrenia /other psychotic disorders Yes**	**Medical record schizophrenia /other psychotic disorders No**	
**Self-reported schizophrenia Yes**	491	124	PPV*: 0.772
**Self-reported schizophrenia No**	1853	332852	NPV*: 0.995
	Sensitivity: 0.209	Specificity: 0.9996	

Positive predictive values (PPV), negative predictive values (NPV), sensitivity and specificity of individuals who self-reported schizophrenia either verbally to a nurse on the initial assessment or on the mental health questionnaire and had a medical record diagnosis of schizophrenia or other psychotic disorders. Other psychotic disorders include codes corresponding to schizotypal disorder, persistent delusional disorders, acute and transient psychotic disorders, induced delusional disorder, schizoaffective disorders, other nonorganic psychosis and unspecified nonorganic psychosis. * Adjusted values based on point prevalence (unadjusted PPV = 0.798374, unadjusted NPV = 0.9944638).

Supplementary Table 5: Diagnosis variables used for self-reported, research interview, and medical record diagnosis groups.

**Array**	**Number of CardiffCOGS cases**	**Number of NCMH cases**	**Number of NCMH controls**
GSA	0	981	484
OmniExpress	632	0	0
PsychArray	0	562	265

Number of participants by OmniExpress/PsychArray/GSA array platforms in the genetic subset.

Supplementary Table 6: Number of other psychotic and mood admissions by primary and secondary schizophrenia admission groups

	**UK Biobank field ID**	**Primary schizophrenia admission (0) (N=209)**	**Secondary schizophrenia admission only (1) (N=459)**
Other psychotic related diagnosis	130877, 130879, 130881, 130885, 130887, 130889	64 (31%)	115 (25%)
Mood diagnosis	130891:130903	73 (35%)	164 (36%)

The number (and percentage) of other psychotic related diagnoses and mood diagnoses of participants who had a primary admission of schizophrenia and a secondary admission of schizophrenia. Diagnoses were assigned based on a hospital admission code (40) or hospital admission and other sources code (41).
(See https://biobank.ndph.ox.ac.uk/showcase/label.cgi?id=2405 for further details).

Supplementary Table 7: Other admission diagnoses by primary and secondary schizophrenia admission groups

	**UK Biobank field ID**	**Primary schizophrenia admission (0) (N=93)**	**Secondary schizophrenia admission only (1) (N=233)**
Delirium	130847	2 (2%)	25 (11%)
Cognitive disorders	130837:130843	3 (3%)	15 (6%)
Substance disorders	130855:130873	12 (13%)	28 (12%)
Anxiety related disorders	130905:130911	16 (17%)	44 (19%)
Other psychiatric diagnosis	130913:130991	15 (16%)	32 (14%)

Table 7 shows the number (and percentage) of other mental health diagnoses of participants who did not have either a psychotic related diagnosis or a mood diagnosis by primary and secondary admission groups. Diagnoses were assigned based on a hospital admission code (40) or hospital admission and other sources code (41).
(See https://biobank.ndph.ox.ac.uk/showcase/label.cgi?id=2405 for further details).

Supplementary Figure 1: NCMH self-reported diagnoses

Has a doctor or health professional ever told you that you have any of the following diagnoses? (tick all that apply)

☐ Attention Deficit Hyperactivity Disorder (ADHD)
☐ Autism
☐ Asperger's or other Autism Spectrum Disorder (e.g. Pervasive Developmental Disorder)
☐ Dyslexia
☐ Dyspraxia
☐ Conduct Disorder
☐ Oppositional Defiant Disorder (ODD)
☐ Tic Disorders
☐ Tourette's Disorder
☐ Intellectual Disability (ID) / Learning Disability (LD)
☐ Depression
☐ Bipolar Disorder / Manic Depression
☐ Mania / Hypomania
☐ Schizoaffective Disorder
☐ Psychosis
☐ Schizophrenia
☐ Postnatal Psychosis / Puerperal Psychosis
☐ Postnatal Depression
☐ Anorexia
☐ Bulimia
☐ Obsessive Compulsive Disorder (OCD)
☐ Agoraphobia
☐ Panic Disorder
☐ Phobias
☐ Anxiety
☐ Borderline Personality Disorder
☐ Other Personality Disorder
☐ Post-Traumatic Stress Disorder (PTSD)
☐ Alzheimer's Disease
☐ Other dementia
☐ Alcohol Abuse / Misuse
☐ Other substance Abuse / Misuse (please specify) ____________
☐ Genetic Syndrome (e.g. VCFS) (please specify) ____________
☐ Self-harm or suicide attempts
☐ Other (please specify) ____________

Page 10

List of mental health diagnoses given to participants to choose from in NCMH cohort.

Supplementary Figure 2: Self-reported diagnoses in UK Biobank's mental health questionnaire

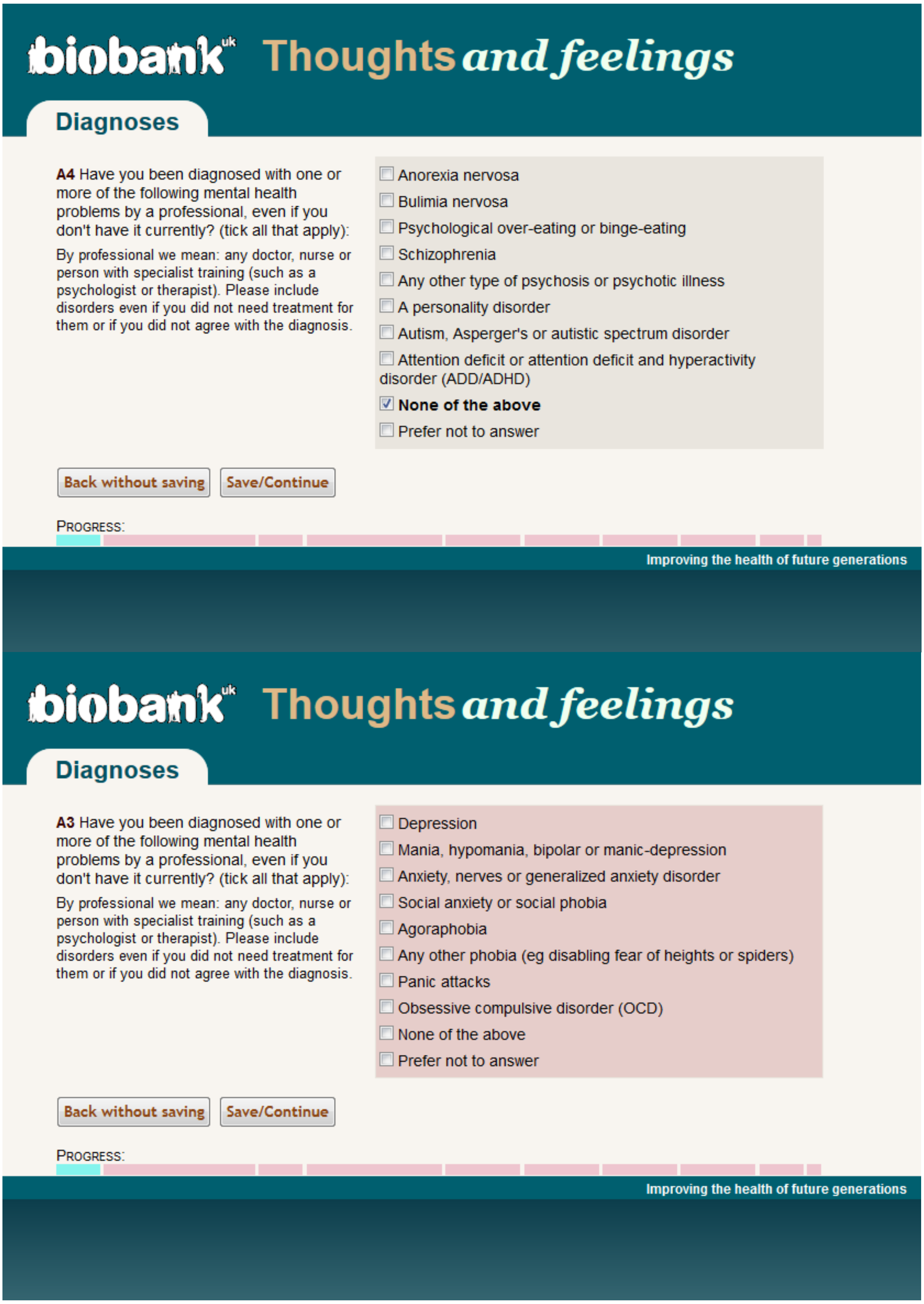
biobank[uk] Thoughts and feelings

Diagnoses

A4 Have you been diagnosed with one or more of the following mental health problems by a professional, even if you don't have it currently? (tick all that apply):

By professional we mean: any doctor, nurse or person with specialist training (such as a psychologist or therapist). Please include disorders even if you did not need treatment for them or if you did not agree with the diagnosis.

- [ ] Anorexia nervosa
- [ ] Bulimia nervosa
- [ ] Psychological over-eating or binge-eating
- [ ] Schizophrenia
- [ ] Any other type of psychosis or psychotic illness
- [ ] A personality disorder
- [ ] Autism, Asperger's or autistic spectrum disorder
- [ ] Attention deficit or attention deficit and hyperactivity disorder (ADD/ADHD)
- [x] **None of the above**
- [ ] Prefer not to answer

Back without saving | Save/Continue

PROGRESS:

Improving the health of future generations

biobank[uk] Thoughts and feelings

Diagnoses

A3 Have you been diagnosed with one or more of the following mental health problems by a professional, even if you don't have it currently? (tick all that apply):

By professional we mean: any doctor, nurse or person with specialist training (such as a psychologist or therapist). Please include disorders even if you did not need treatment for them or if you did not agree with the diagnosis.

- [ ] Depression
- [ ] Mania, hypomania, bipolar or manic-depression
- [ ] Anxiety, nerves or generalized anxiety disorder
- [ ] Social anxiety or social phobia
- [ ] Agoraphobia
- [ ] Any other phobia (eg disabling fear of heights or spiders)
- [ ] Panic attacks
- [ ] Obsessive compulsive disorder (OCD)
- [ ] None of the above
- [ ] Prefer not to answer

Back without saving | Save/Continue

PROGRESS:

Improving the health of future generations

List of diagnoses participants can choose from in the mental health questionnaire (MHQ) in the UK Biobank.

Supplementary Figure 3: Principal components 1-6 by array

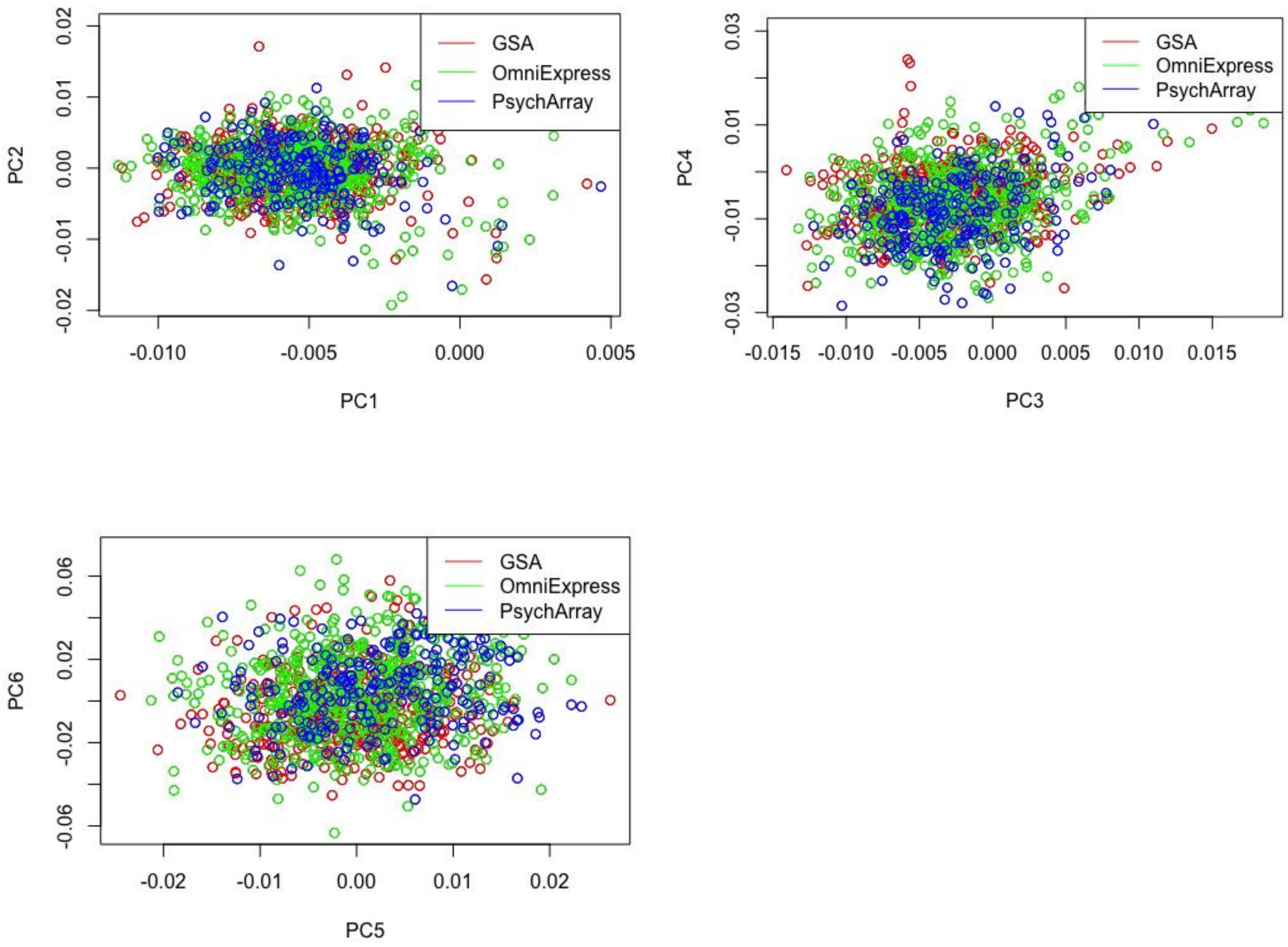

Principal component (PC) plots by each genotyping array (Infinium Global Screening Array (GSA), OmniExpress, PsychArray) to demonstrate any batch effects. The plots demonstrate that the distribution of individuals across PCs did not differ depending on which genotyping array was used.

Supplementary Figure 4: Diagnosis endorsements and schizophrenia PRS

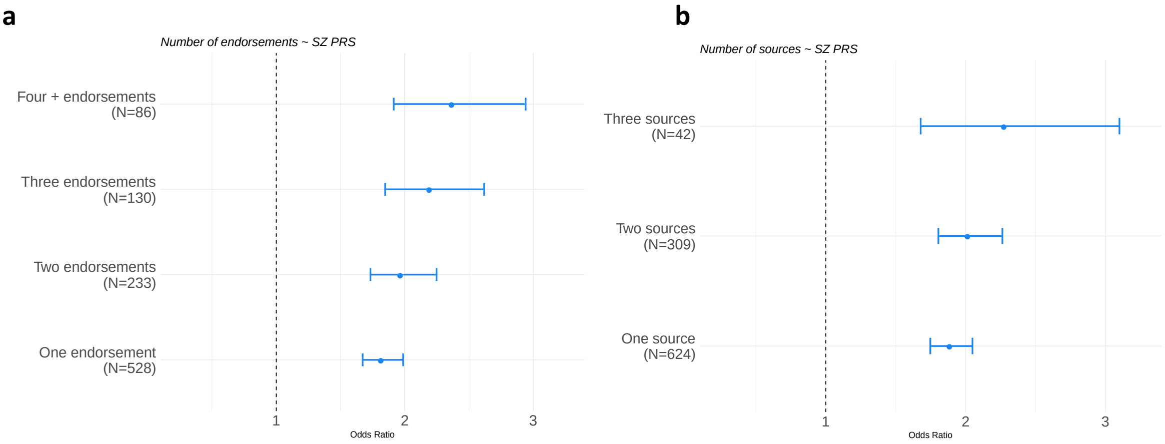

As participants' schizophrenia PRS increased, the number of times a diagnosis was reported increased. Figure 5a displays the number of endorsements (number of times a diagnosis is reported in UK Biobank). Figure 5b shows the same graph when concatenating all hospital admissions into one hospital endorsement.

Supplementary Figure 5: Hospital admissions for schizophrenia and schizophrenia PRS

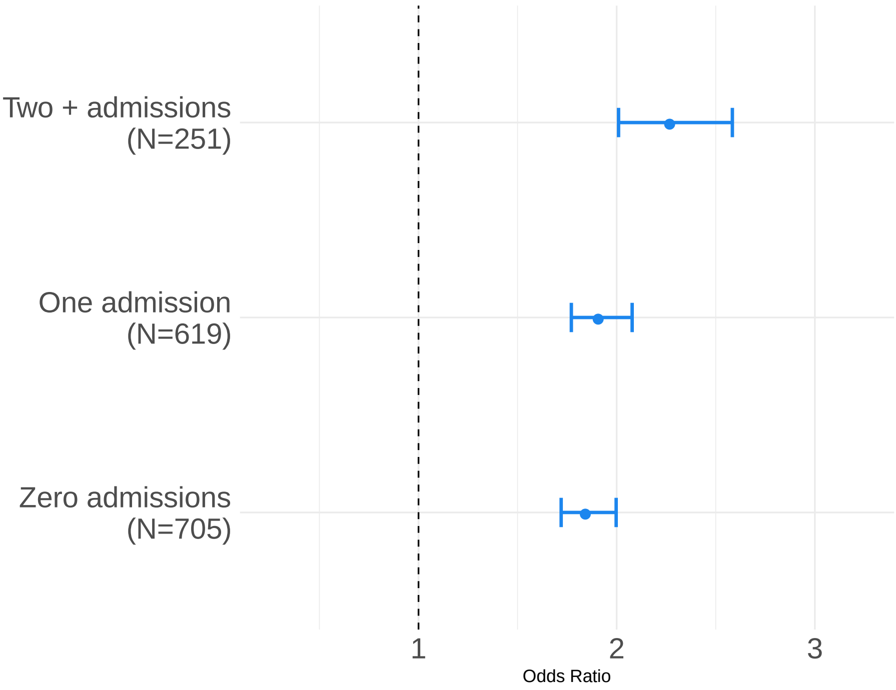

As participants' schizophrenia PRS increases the odds of having an admission increases.

Supplementary Figure 6: Schizophrenia PRS and primary and secondary admission groups

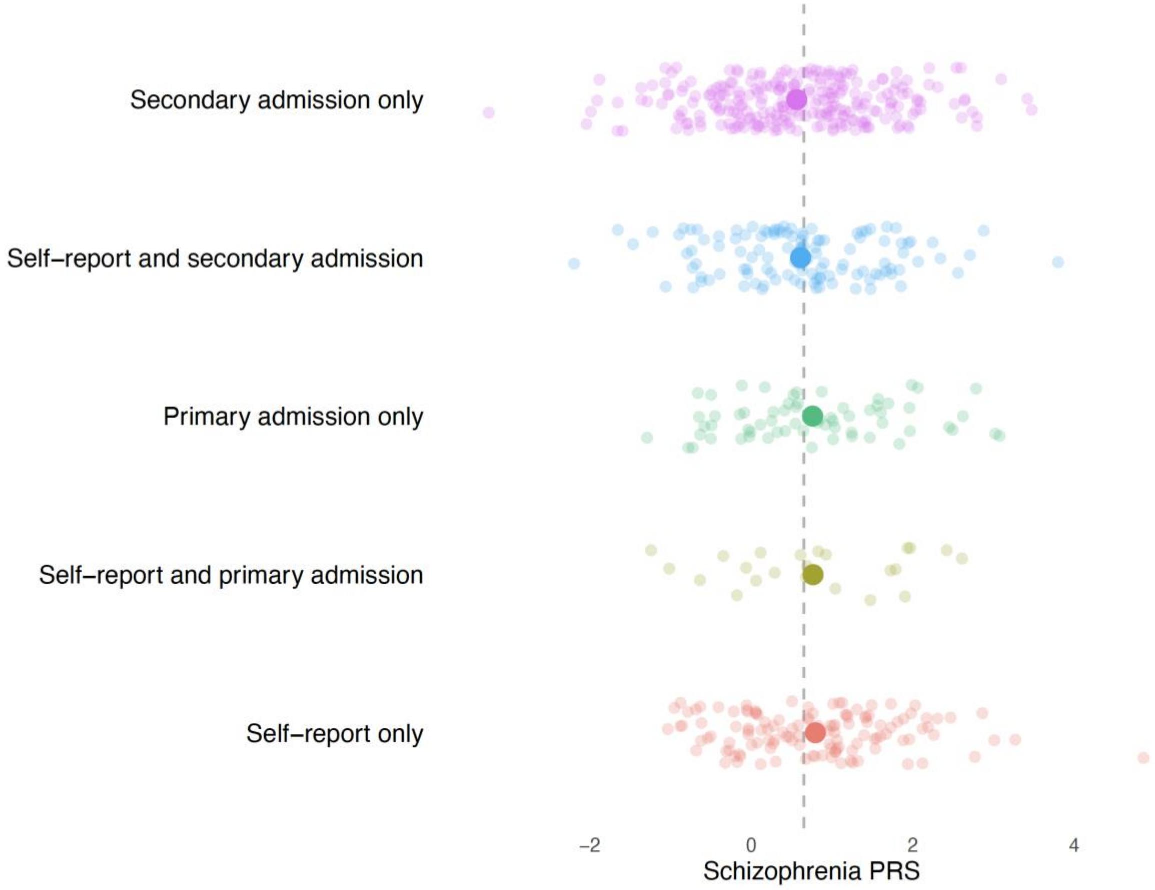

Schizophrenia PRS by primary and secondary admission diagnoses, subdivided into those with only an admission code and those with both a self-report and admission code. The dotted line represents the mean schizophrenia polygenic risk score.

Supplementary Note 1: PPV/NPV prevalence adjustment

In the UK Biobank, predictive values were adjusted to the point prevalence of schizophrenia (0.6%). The following formulas were used to adjust the PPV and NPV:

PPV = (sensitivity x prevalence)/[(sensitivity x prevalence)+((1 – specificity) x (1 – prevalence))]

NPV = (specificity x (1 – prevalence))/[(specificity x (1 – prevalence))+((1 – sensitivity) x prevalence)]
